# Label-Free Detection of 2,4-Dinitrotoluene Using a Laser-Induced Graphene Based Chemiresistive Sensor

**DOI:** 10.1021/acsomega.5c12391

**Published:** 2026-02-05

**Authors:** Seda Kol, Mehmet Sezer, Fatmanur Kocaman Kabil, Ersin Kayahan, Ahmet Yavuz Oral

**Affiliations:** † Department of Materials Science and Engineering, 52962 Gebze Technical University, Kocaeli 41400, Turkey; ‡ Institute of Nanotechnology, Gebze Technical University, Kocaeli 41400, Turkey; § Department of Electrical and Electronics Engineering, Erzurum Technical University, Erzurum 25100, Turkey; ∥ Laser Technologies Research and Application Center (LATARUM), 52980 Kocaeli University, Kocaeli 41275, Turkey

## Abstract

The rapid and sensitive detection of nitroaromatic explosives is of paramount importance for both security and environmental monitoring. In this study, a label-free chemiresistive sensor based on laser-induced graphene (LIG) was developed for the selective detection of 2,4-dinitrotoluene (DNT). LIG films were directly fabricated on polyimide substrates via a single-step laser writing process, resulting in porous and conductive surfaces without additional modification. The structural, chemical, and electrical properties of the fabricated materials were comprehensively evaluated using scanning electron microscopy (SEM), X-ray diffraction (XRD), Raman spectroscopy, Fourier transform infrared (FTIR) spectroscopy, X-ray photoelectron spectroscopy (XPS). The electrical properties were characterized by current–voltage (*I*–*V*) measurements using a Kelvin (pseudofour-point) configuration. SEM revealed a porous morphology formed during laser scribing, while XRD and Raman spectroscopy confirmed multilayer graphene (∼5 layers) with relatively low defect density. FTIR spectroscopy indicated residual oxygen-containing functional groups, and XPS verified DNT adsorption. The fabricated films exhibited a uniform electrical conductivity of 1545 S/m. By employing these films, a chemiresistive sensor was developed, which demonstrated a response toward DNT, achieving an estimated detection limit (LOD) of 3.79%, corresponding to 2.4 × 10^–9^ M. Strong selectivity was observed against structurally related interferents such as nitrotoluene, toluene, and ethanol. These results demonstrated that LIG-based flexible sensors provide a low-cost, scalable, and selective platform for explosive detection with promising applications in security and environmental monitoring.

## Graphical abstract

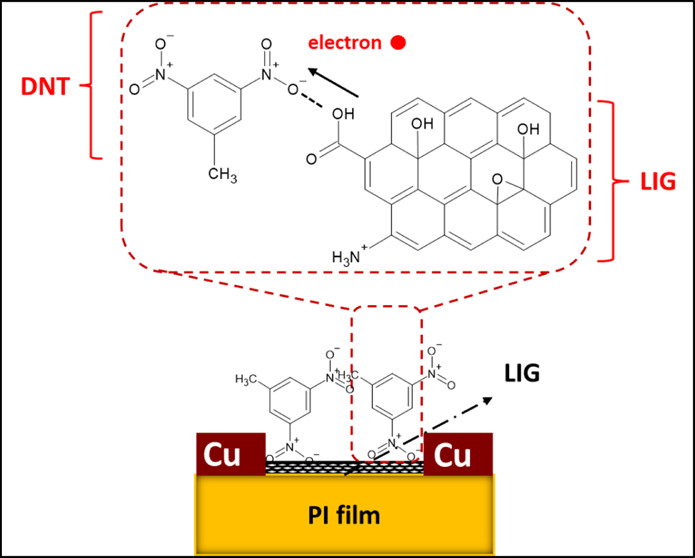

## 1 Introduction

Terrorism has long-term social, economic, and political consequences, but it has also driven global cooperation and security policies.^1^ Since explosives are present in various environments, their rapid and sensitive detection is critical in locations such as minefields, ammunition depots, transport hubs, and blast sites to ensure safety and security,^2^ highlighting the urgent need for reliable sensors. 2,4-Dinitrotoluene (DNT), a byproduct or intermediate generated during the production of TNT, is an explosive material. The detection of nitroaromatic compounds (NACs) such as DNT and TNT is challenging, as TNT has a saturated vapor pressure of only 4.8 × 10^–6^ Torr at room temperature. This necessitates the development of highly sensitive sensor systems capable of detecting explosives even at trace levels.^3–6^

Today, various techniques are employed for the detection of 2,4-dinitrotoluene (DNT) and other nitroaromatic explosives, including ion mobility spectrometry (IMS),^7^ gas chromatography–mass spectrometry (GC–MS),^8^ quartz crystal microbalance (QCM),^9^ Raman and FTIR spectroscopy^10^ and electrochemical sensing^11^ platforms. Many of these methods are capable of detecting nitroaromatic compounds at very low levels, typically in the ppb range and down to ng-pg amounts under laboratory conditions.^7–11^ IMS-based systems are widely used in security screening applications and can achieve detection limits at low ppb or even ppt levels for vapor-phase nitroaromatics. However, their performance is highly susceptible to environmental factors, and the requirement for frequent calibration limits their practical applicability.^7^ GC–MS, which is considered the gold standard for explosive analysis, provides excellent sensitivity down to low ppb or ng levels. Nevertheless, its high cost, bulky and nonportable instrumentation, long analysis times, and dependence on carrier gases restrict its use in rapid and on-site detection scenarios.^8^ QCM-based sensors can achieve ppb-level sensitivity by monitoring mass changes induced by analyte adsorption. However, their response is strongly influenced by variations in humidity and temperature, which hampers long-term stability under field conditions.^9^ Vibrational spectroscopic techniques such as Raman and FTIR spectroscopy enable reliable identification through molecular fingerprint information on nitroaromatic compounds. However, trace-level detection generally requires higher analyte concentrations or advanced optical setups.^10^ Electrochemical detection approaches, which exploit the redox activity of nitro groups, have demonstrated detection limits ranging from subppm to ppb levels for DNT in solutions. However, these methods are predominantly based on liquid-phase analysis and often involve additional steps, such as electrode preparation or sample pretreatment.^11^

However, despite offering high sensitivity, these methods have certain limitations such as high cost, complex instrumentation, limited portability, and lack of field applicability. Therefore, the development of alternative sensor technologies that are low-cost, portable, fast responding, and easy to use is of great importance.^12^ Today, sensors developed for the detection of TNT-based explosives are composed of receptor-based structures that incorporate materials such as metallic nanoparticles (e.g., silver (Ag), gold (Au), platinum (Pt)), porous silicon (porous Si), indium tin oxide (ITO), and various organic polymers.^13^ However, such sensors often suffer from disadvantages such as low selectivity, limited sensitivity, and slow response times, which has increased the demand for next-generation materials.

Graphene is a two-dimensional material composed of a single layer of carbon atoms arranged in a honeycomb lattice and held together by strong sp^2^ covalent bonds. Its electrical conductivity can approach or surpass that of copper (∼10^8^ S/m in theory), and it possesses a large specific surface area of approximately 2630 m^2^/g. Additionally, it demonstrates remarkable elasticity, with the ability to sustain tensile strains of up to 20–25% before mechanical failure. These exceptional properties make graphene an ideal material for flexible, lightweight, and high-performance sensor applications.^14–17^ In this context, laser-induced graphene (LIG), synthesized by directly writing laser beams onto polymer surfaces, has emerged as a faster, scalable, and cost-effective alternative to conventional graphene production methods. This single-step, mask-free process enables the fabrication of flexible chemiresistive sensor platforms without complex manufacturing steps.^18^ LIG is most commonly produced by direct CO_2_ laser writing onto aromatic polymers such as polyimide (PI).^19,20^

Recent studies have demonstrated the successful application of laser-induced graphene (LIG)-based sensors for detecting gases such as ammonia, acetylene, NO_2_, H_2_S, and various volatile organic compounds (VOCs), leveraging LIG’s large surface area, high conductivity, and 3D porous structure.^21–24^ Current research focuses on enhancing sensor stability, portability, scalable fabrication, and real-world applicability. Flexible LIG platforms offer cost-effective, mass-producible, and durable solutions, with validation in real samples bridging the gap between lab-scale and practical use. Advances in LIG-based electrochemical sensors increasingly rely on hybrid interface engineering, incorporating functional nanomaterials—such as metal oxide nanoparticles, metal–organic frameworks (MOFs), and polymer-supported composites—to boost electrocatalytic activity, sensitivity, and signal stability.^25–27^ For example, NiFe_2_O_4_ and CeO_2_ nanoparticles improved Mn^2+^ detection by introducing active sites and facilitating electron transfer.^25^ Likewise, sensors commodified with polyaniline-functionalized Fe_3_O_4_ and Ce-based MOFs enabled ultrasensitive detection of Cr­(VI) in aqueous environments.^26^ Ag and TiO_2_ nanoparticles, dispersed via polymeric matrices, also enhanced antibiotic detection by increasing catalytic efficiency.^27^

The detection of nitroaromatic explosives using high-performance LIG-based sensors typically relies on additional functional layers. For example, pseudomolecularly imprinted poly­(3,4-ethylenedioxythiophene)/LIG (MIPEDOT/LIG) sensors have been developed by incorporating polymeric recognition layers onto the LIG surface to form selective binding sites. Such systems enable the detection of multiple nitroaromatic explosives such as TNT, TNP, DNT, TNB, DNP, and DNB at ppb levels, and this high sensitivity and selectivity directly stems from the molecular imprinting strategy.^28^ However, this approach complicates sensor design due to additional chemical synthesis steps, template removal processes, and multistage manufacturing requirements, making it difficult to directly evaluate the intrinsic sensing capability of LIG. Current LIG-based explosive sensors often depend on biorecognition elements, which limit stability, scalability, and cost-efficiency. However, label-free detection approaches remain underexplored. Developing functionalization-free LIG platforms for direct DNT detection can address these limitations and simplify fabrication.^29^

The main objective of this study was to evaluate the intrinsic sensing capability of pristine laser-induced graphene (LIG) films for the label-free detection of 2,4-dinitrotoluene (DNT) by characterizing their chemiresistive response under varying analyte concentrations, without employing any surface modification or biorecognition elements. LIG films were fabricated via direct laser writing and used as sensing platforms without further chemical treatment. This label-free approach offers advantages such as simplified fabrication, reduced cost, and improved stability by eliminating the need for fragile and expensive recognition layers like peptides or antibodies. To the best of our knowledge, direct detection of nitroaromatic compounds using unmodified LIG-based sensors has not been previously reported. This work addresses a significant gap by demonstrating the feasibility of using pristine LIG for DNT sensing in a surface-functionalization-free manner.

## 2 Materials and Methods

### 2.1 Chemicals and Materials

PI (Kapton Polyimide Film) with 125 μm thickness, used for the laser-induction process, was purchased from Yatiz Elektrik. Toluene for analysis grade was purchased from Merck (catalog no. 108325). Absolute ethanol was obtained from Merck (catalog no. 100983). 2-Nitrotoluene was purchased from Sigma-Aldrich (catalog no. 101397). 2,4-Dinitrotoluene (DNT, 97% purity) was obtained from Sigma-Aldrich.

### 2.2 Fabrication of Laser-Induced Graphene

LIG was fabricated using a commercially available PI (polyimide) film and a VLS 6.60 Universal Laser system (wavelength: 10.6 μm). The laser system is equipped with a CO_2_ laser generator and has a maximum power capacity of 40 W. LIG was produced in a single-direction scan mode (from left to right) using 12.5% laser power. The resulting LIG patterns were 10 mm × 10 mm in size. A schematic illustration of the fabrication process is shown in Figure 1.

**Figure 1:**
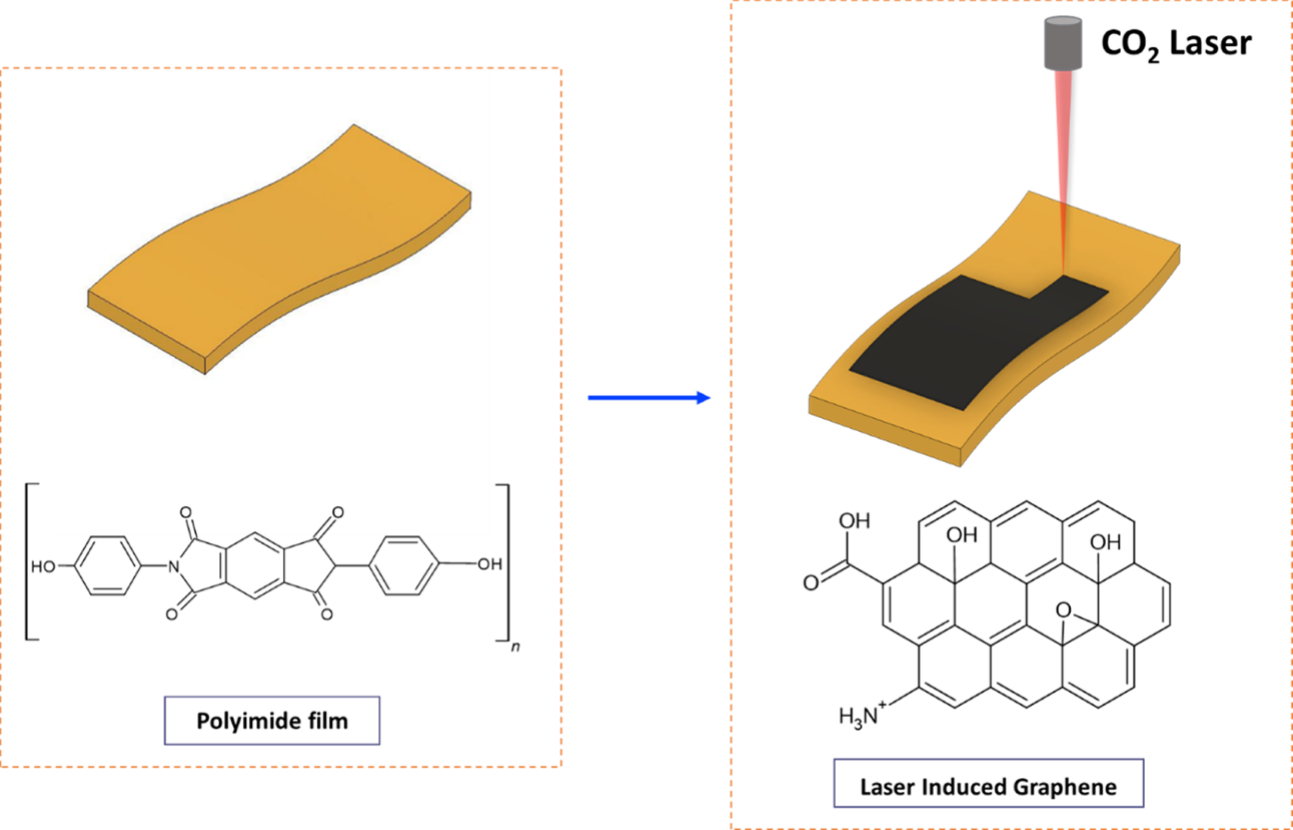
Schematic illustration of the LIG formation process on polyimide (PI) film.

### 2.3 Preparation of DNT Solutions

2,4-Dinitrotoluene (DNT, *M*_r_ = 182.14 g·mol^–1^) stock and working solutions were prepared in ethanol. A **0.10 M** stock was made by dissolving **1.82 g** of DNT in a **100 mL** volumetric flask and filling to the mark with ethanol. Working solutions were obtained by serial dilutions as follows:

- **1.0 × 10** ^**–3**^ **M**: 1.00 mL of 0.10 M + 99.00 mL ethanol (1:100).
- **1.0 × 10** ^**–6**^ **M**: 1.00 mL of 1.0 × 10^–3^ M + 999.00 mL ethanol (1:1000).
- **1.0 × 10** ^**–9**^ **M**: 1.00 mL of 1.0 × 10^–6^ M + 999.00 mL ethanol (1:1000).

Before use, all solutions were magnetically stirred to ensure homogeneity. A fixed volume of 10 μL was drop-cast onto the LIG surface, allowed to evaporate, and the electrical response was recorded.

### 2.4 Characterization Techniques

The surface and cross-sectional morphologies of the samples were examined using a Philips XL 30 SFEG SEM. Prior to imaging, all samples were coated with a thin layer of gold via sputtering to minimize charging and improve image quality at high magnification. The crystalline structures of LIG samples were analyzed using a Rigaku *D*-max RINT 2200 diffractometer with Cu Kα radiation (15°–50°, 0.2°/min).

Raman spectra were obtained using a Renishaw Virsa system with 532 nm laser excitation at room temperature. FTIR spectra were recorded using a Bruker Tensor 27 in the range of 4000–500 cm^–1^. For FTIR analysis, the LIG layer was gently scraped from the PI substrate, mixed with KBr, and pressed into pellets to identify oxygen-containing functional groups formed during laser conversion. XPS measurements were carried out using Phoibos 100, SPECS GmbH to determine surface chemical composition and bonding states. Survey and high-resolution spectra were analyzed via CasaXPS software. Electrical resistance measurements were performed using a Gamry Reference 3000 electrochemical workstation with a Kelvin (pseudofour-point) connection.

### 2.5 Electrical Measurement Scheme of the Sensors

The LIG surface on a PI substrate was selected as the recognition surface for flexible sensors developed for the detection of DNT-based explosives. For resistance tests of DNT and other analytes, LIG films with dimensions of 2 × 1 cm were produced. Electrical connections were made using copper wires, and the contacts were secured with copper tape. The measurement setup and the way the sample is connected to the system are illustrated in Figure 2.

**Figure 2:**
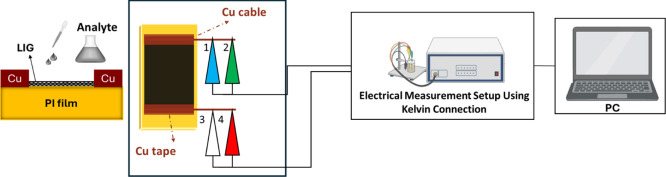
Electrical configuration of the LIG sample prepared for resistance measurements.

#### 2.5.1 Mean Resistance (*R̅*)

The arithmetic means of all resistance values

1 $$\mathrm{R̅}=\frac{\sum_{i=1}^{n}R_{i}}{n}$$

#### 2.5.2 Standard Deviation

Indicates how much the resistance values deviate from the mean

2 $$SD=\sqrt{\frac{\sum_{i=1}^{n}{\left(R_{i}-\mathrm{R̅}\right)}^{2}}{n-1}}$$

To evaluate the electrical changes due to DNT molecule adsorption, the response (*T*_DNT_ or *T*_TNT_) was calculated in three steps. First, the initial resistance (*R*_0_) of the LIG sensor was measured before analyte exposure. Then, after applying the DNT solution to the sensor surface and allowing it to absorb, the final resistance (*R*_final_) was recorded. The change in resistance (Δ*R*) was calculated using the equation

3 $$\Delta R=R_{final}-R_{0}$$

#### 2.5.3 Response (*T*_R_ %)

The fractional change in resistance relative to the initial value, expressed as a percentage

4 $$T_{R}\;\%=\frac{\Delta R}{R_{0}}x100$$

#### 2.5.4 Limit of Detection Calculation

The limit of detection (LOD) is the lowest analyte concentration or amount that can be reliably detected by a sensor or analytical instrument. To determine the limit of detection (LOD), the standard deviation of the background signal was first determined using blank samples -solvent without analyte- (ethanol in our case, without DNT). The LOD was then calculated using this value

5 $$LOD=T_{0}+3S_{0}$$

In this equation, *T*_0_ represents the baseline signal obtained from blank samples containing no DNT, while *S*_0_ denotes the standard deviation of this baseline signal.

#### 2.5.5 Selectivity Calculation Method (*S*)

Selectivity (*S*) indicates the sensor’s preferential sensitivity toward a specific substance (e.g., DNT) over other reference analytes (e.g., nitrotoluene, toluene, ethanol). Selectivity is calculated using

6 $$S=\frac{T_{R,analyte}}{T_{R,DNT}}$$

According to this definition,

- *S* = 1 indicates equal sensitivity to DNT and the reference analyte.
- *S* < 1 indicates preferential sensitivity toward DNT.
- *S* > 1 indicates the sensor is more sensitive to the reference analyte, which is undesirable.^30^

## 3 Results and Discussion

### 3.1 Structural, Chemical, and Electrical Characterization of LIG

Scanning electron microscopy (SEM) analysis was performed to characterize the surface morphology of the LIG films (Figure 3a,b). Low- and high-magnification SEM images reveal the surface features of the LIG structures. At low magnification, periodic laser scan tracks are observed on the surface at approximately 50 μm intervals, corresponding to the raster pattern of the laser (Figure 3a). High-magnification images show a foamy and porous morphology, attributed to the rapid release of gases during the laser scribing process (Figure 3b). This porosity increases the effective surface area, which is particularly beneficial for sensor applications.

**Figure 3:**
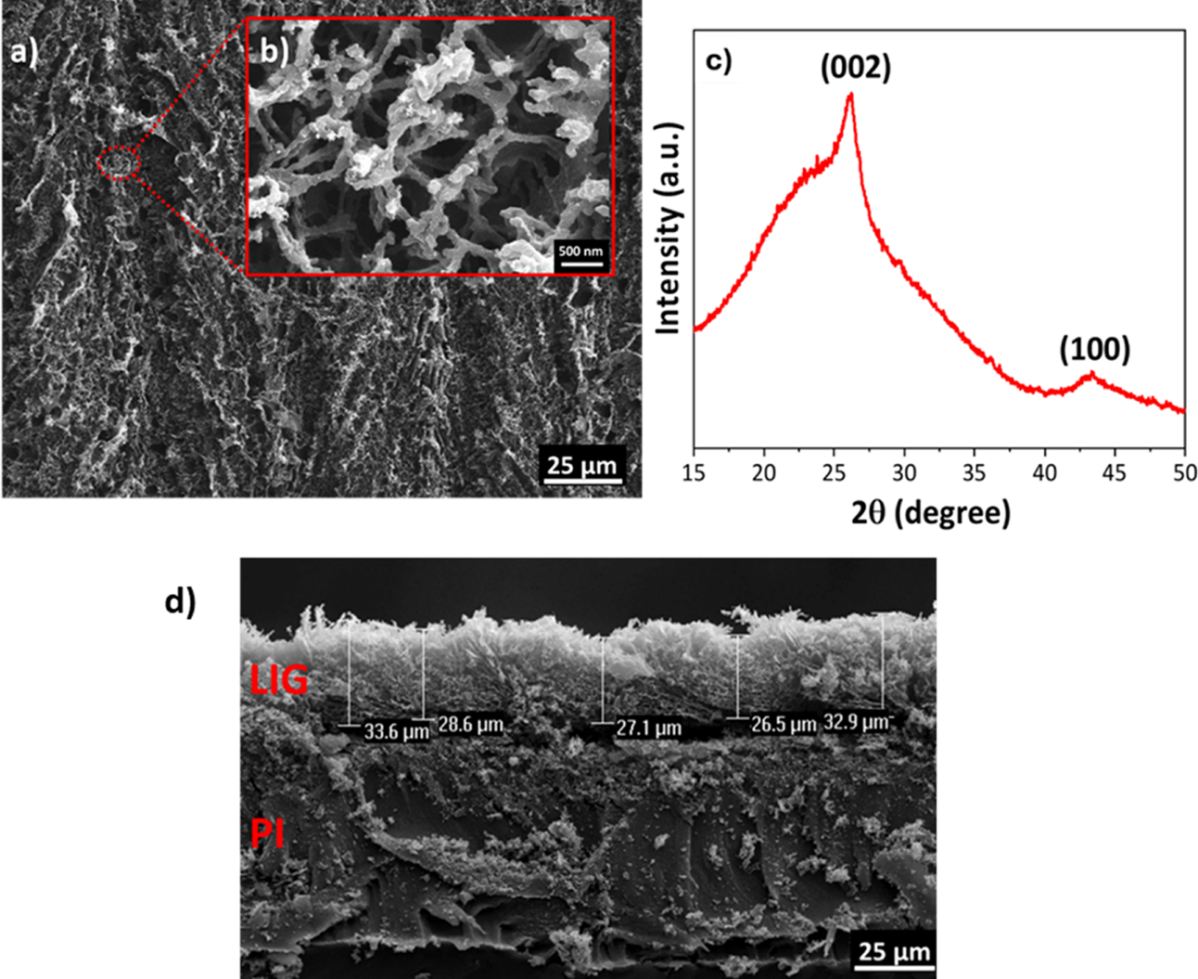
SEM images of the LIG film at (a) low and (b) high magnifications, (c) XRD pattern of the powders scraped from the LIG film, and (d) cross-sectional SEM image.

The crystal structure of the LIG powder scraped from the PI film was investigated by X-ray diffraction (XRD) using Cu Kα radiation (λ = 1.5406 Å). As shown in Figure 3c), two characteristic reflections of graphene-based materials were observed: the (002) plane at 2θ = 26.1°, corresponding to an interlayer spacing (*d*_002_) of approximately 3.4 Å, and the (100) plane at 2θ = 43.1°, associated with in-plane ordering, yielding a *d*-spacing of about 2.09 Å. The average crystallite thickness along the *c*-axis (*L*_c_) was determined using the Scherrer equation^31^

7 $$L_{C}=\frac{K\lambda }{\beta \cos\;\theta }$$

where *K* is the shape factor (typically 0.9), λ is the X-ray wavelength, β is the full width at half-maximum (fwhm) of the diffraction peak (in radians), and θ is the Bragg angle. From the calculated *L*_c_ and the interlayer spacing (*d*_002_), the average number of graphene layers was estimated using

8 $$N=\frac{L_{C}}{d_{002}}+1$$

Based on this approach, the number of stacked graphene layers in the LIG powder was calculated to be approximately 5.3.

Figure 3d presents a cross-sectional SEM image showing the LIG layer formed on the PI substrate, with a measured thickness of approximately 29.7 μm.

Figure 4a presents the Raman spectrum of the PI before being exposed to laser irradiation. A breathing mode corresponding to the simultaneous expansion and contraction of aromatic rings is observed at 1122 cm^–1^. Stretching vibrations of C–N–C bonds appear at 1390 cm^–1^ and vibrations associated with aromatic C=C bonds are seen around 1600 cm^–1^. Additionally, a vibrational band corresponding to the carbonyl (C=O) group is detected at 1789 cm^–1^. These peaks reflect the characteristic chemical bonds specific to the polyimide structure.^32^ In the Raman spectrum of LIG, the D band at approximately 1350 cm^–1^ and the G band at 1580 cm^–1^ are clearly observed (Figure 4b). The calculated *I*_D_/*I*_G_ ratio is 0.55 indicating that the graphene possesses a relatively low defect density. These findings demonstrate that the polyimide structure was successfully converted into graphene through laser processing.^33^ The 2D band, which plays a critical role in determining whether the graphene is single-layer or multilayer, appears broad and of low intensity around 2700 cm^–1^. Based on the peak intensities corresponding to these bands, the *I*_2D_/*I*_G_ ratio was calculated to be approximately 0.51. According to the literature, graphene with an *I*_2D_/*I*_G_ ratio in the range of 0.3–0.7 is typically classified as multilayer graphene, with a thickness corresponding to approximately 5–7 layers.^34^ Multilayer structure identified by Raman analysis is consistent with the XRD results.

**Figure 4:**
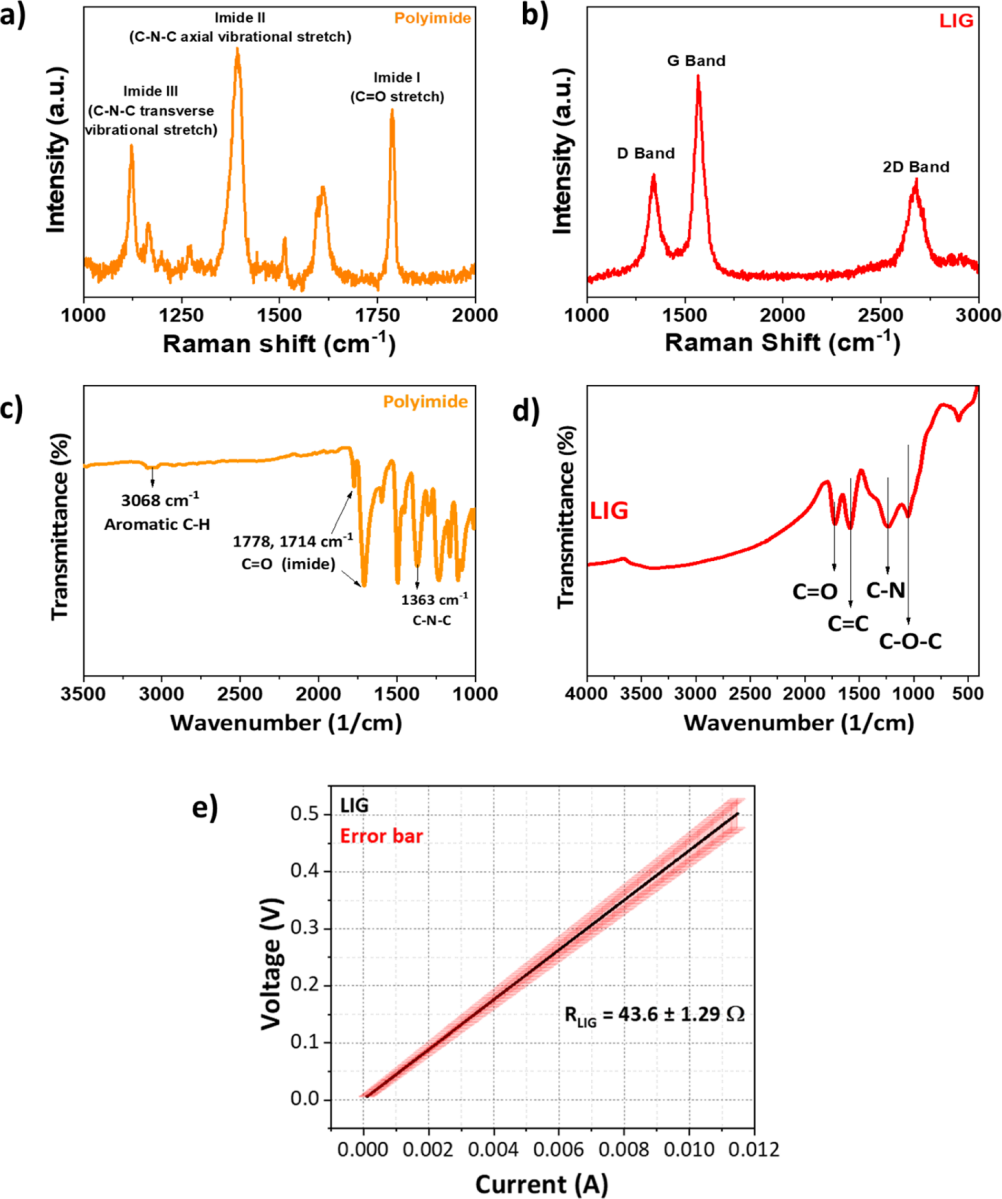
Raman spectra of (a) polyimide and (b) the LIG film; FTIR spectra of (c) polyimide and (d) the LIG film; and (e) *I*–*V* curve of the LIG film measured using the Kelvin (pseudofour-point) connection method.

Figure 4c presents the Fourier transform infrared spectroscopy (FTIR) spectrum of the PI film. The peak observed around 3068 cm^–1^ corresponds to the C–H stretching vibrations of aromatic rings. The distinct peaks at 1778 cm^–1^ and 1714 cm^–1^ are attributed to the characteristic imide carbonyl (C=O) groups within the polyimide backbone. Additionally, the peak at 1363 cm^–1^ corresponds to the C–N–C stretching vibration in the imide ring.^35^ Figure 4d displays the FTIR spectrum of the LIG film. In the LIG spectrum, the sharp peak at 1726 cm^–1^ corresponds to carbonyl (C=O) groups, while the peak at 1583 cm^–1^ is attributed to aromatic C=C bonds. The peak observed at 1244 cm^–1^ is related to C–N and/or C–O stretching vibrations, and the peak at 1049 cm^–1^ is associated with epoxide (C–O–C) or hydroxyl (C–OH) groups.^36^

Raman and FTIR analyses collectively confirm the successful conversion of polyimide to laser-induced graphene, with Raman providing insights into the graphitic structure and defect density, while FTIR reveals the presence of oxygen-containing functional groups, such as carbonyl, hydroxyl, and epoxy, indicating partial oxidation on the LIG surface.

To evaluate the electrical properties of the fabricated LIG films, resistance measurements were performed using a Gamry Reference 3000 electrochemical workstation. A Kelvin connection (pseudo four-point probe) configuration, with two wires connected to each contact point, was employed to minimize contact resistance. The intrinsic resistance of bare LIG films was determined according to Ohm’s law (*V* = *IR*), with resistance values calculated from the slope of the current–voltage (*I*–*V*) curves shown in Figure 4e.

*I*–*V* characteristics of eight identically prepared LIG films were recorded by varying the applied voltage from 0 to 0.5 V with a step size of 2 mV. A fully linear relationship between current and voltage was observed, and average resistance values were derived from the slopes. The standard deviation was obtained from the variation among these slope-derived values, and the corresponding error band was included in the graphical representation.

Statistical analysis across different regions of the film surface revealed an average resistance of 43.62 Ω with a standard deviation (SD) of ±1.29 Ω, based on three independent measurements from each film. This analysis highlights the spatial uniformity of film conductivity.

For chemiresistive sensing applications, high substrate conductivity is essential to enable the rapid and sensitive detection of electrical signals. The electrical conductivity (σ) of the LIG films was calculated using the equation $\sigma =\frac{L}{R\times A}$ , where *L* is the electrode spacing, *R* is the measured resistance, and *A* is the cross-sectional area of the LIG strip (*A* = *w* × *t*). From the *I*–*V* measurements (Figure 4e), the resistance of the LIG films was determined to be 43.62 ± 1.29 Ω. Based on an electrode spacing of 2 cm, a channel width of 1 cm, and the LIG thickness obtained from the cross-sectional SEM images (Figure 3d), the electrical conductivity was calculated to be 1545 S/m.

Nitroaromatic compounds such as DNT can interact strongly with conductive sensing surfaces due to the pronounced electron-withdrawing properties of the nitro groups (−NO_2_) in their structure. These nitro groups exhibit charge-transfer behavior, facilitating electron exchange at the sensor surface, which generates a measurable electrical response. Rather than involving a chemical transformation, the sensing process is governed by adsorption-induced interactions between the nitroaromatic molecules and the sensing material. The efficiency and magnitude of this interaction depend on factors such as the electrical conductivity, surface morphology, and surface electronic properties of the sensing material. The fundamental working principle of chemiresistive sensors for the detection of nitroaromatic compounds relies on the strong electron-accepting character of these electron-deficient nitro groups on the sensing material.^37^

High-resolution N 1s XPS analysis confirmed the chemical adsorption of DNT molecules onto the LIG surface. As shown in Figure 5a, the spectrum of bare LIG exhibited no distinct nitrogen-related peaks, whereas LIG exposed to 0.1 M DNT displayed additional deconvoluted peaks as shown in Figure 5b. Among them, the pronounced feature at 406.4 eV was assigned to nitro nitrogen (−NO_2_) groups, serving as a direct chemical signature of DNT. The observed binding energy is consistent with literature values reported for nitroaromatic adsorption on carbon-based surfaces,^38^ confirming that the signal arises from DNT adsorption rather than background nitrogen. The strong electron-withdrawing character of nitro groups facilitates charge transfer interactions with the LIG surface, which in turn leads to measurable resistance changes. These XPS findings not only corroborate the proposed binding mechanism but also establish a clear correlation between molecular adsorption and the sensor’s electrical response.

**Figure 5:**
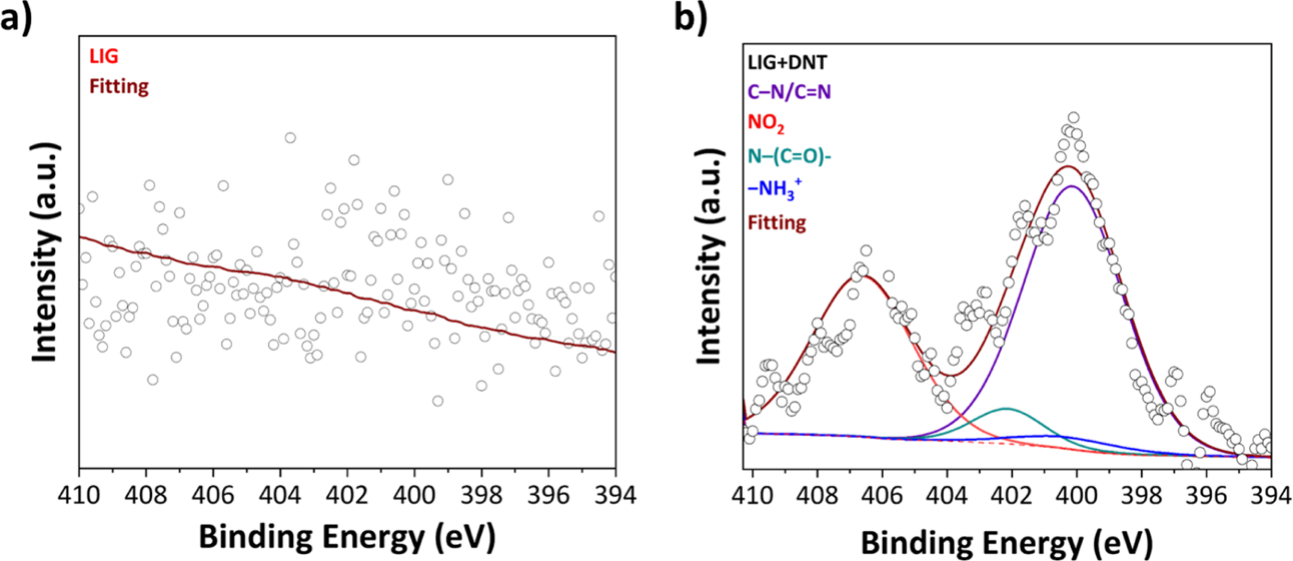
N 1s high resolution XPS spectra of (a) bare LIG and (b) DNT + LIG.

### 3.2 Performance Evaluation of the Sensor

#### 3.2.1 Determination of Limit of Detection

A wide concentration range was selected to evaluate the sensor’s linearity and chemiresistive response across varying analyte levels. Since no prior studies using label-free LIG platforms for DNT detection exist, it was essential to scan a broad range to determine the approximate location of the limit of detection (LOD). To achieve this without exhaustive testing, a limited number of strategically chosen concentrations representing low, medium, and high levels were employed, ensuring comprehensive spectral coverage while balancing data acquisition efficiency. For the ethanol blank (without any target analyte), the mean resistance change obtained from three repeated measurements was $\overset{®}{R_{eth}}=44.37\;\Omega$ , calculated using eq 1. Based on this average value, the initial response *T*_0_ % was determined using eq 4 and calculated to be 1.77. The standard deviation of the measurements was determined using eq 2 as *S*_0_ = 0.293 Ω. When the initial resistance *R*_0_ was taken as the reference, the percentage standard deviation was obtained as *S*_0_ % = 0.673. Using these parameters, the limit of detection (LOD) was calculated using eq 5 to be 3.79%.

Figure 6a shows the response (*T*_R_ %) observed on LIG surfaces when exposed to varying DNT concentrations (0.1 M, 10^–3^ M, 10^–6^ M and 10^–9^ M). The results clearly show that the electrical resistance of the LIG surfaces increases with the applied DNT concentration. The highest resistance changes of 18.92% was recorded at 0.1 M. This was followed by 12.11% and 10.11% at 10^–3^ M and 10^–6^ M, respectively. At the lowest concentration (10^–9^ M), the resistance change was measured as 2.72%.

**Figure 6:**
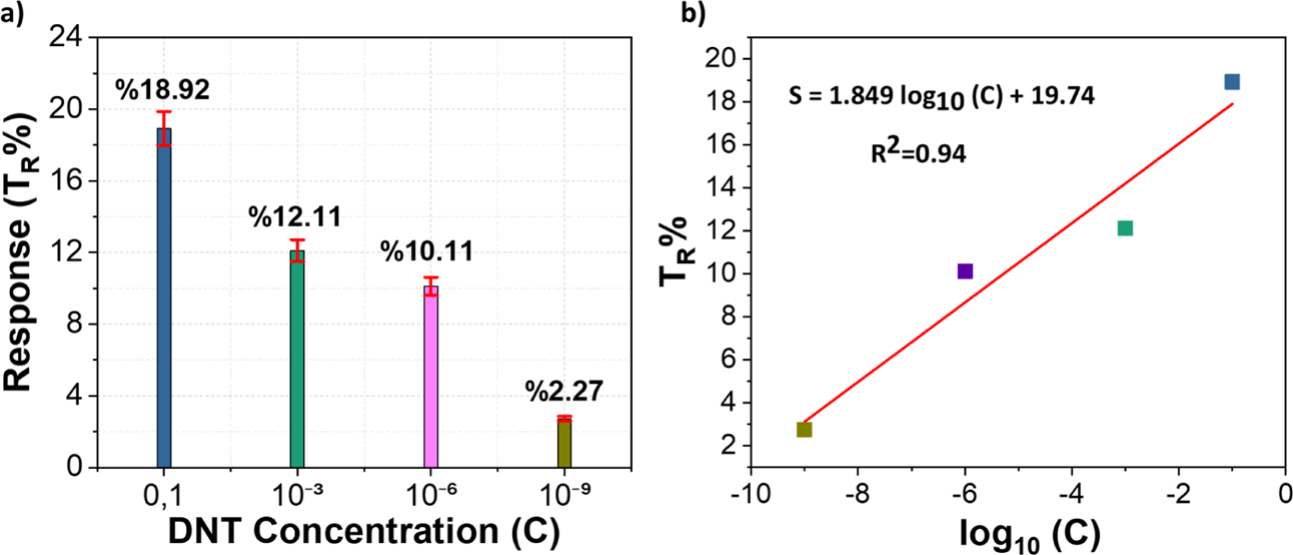
(a) Response (TR %) of the LIG sensor to different DNT concentrations, with error bars representing the standard deviation of three independent measurements. (b) Calibration curve showing the linear relationship between the sensor response (TR %) and the logarithm of the DNT concentration (log_10_ *C*).

Calibration of the LIG sensor was performed using these four DNT concentrations. The sensor response (*T*_R_ %) was plotted as a function of the logarithm of the analyte concentration (log_10_ *C*), as shown in Figure 6b. A linear regression was applied to the data, yielding eq 9.

9 $$T_{R}\;\%=1.849\log_{10}\left(C\right)+19.74$$

By substituting the LOD response value (3.79%) into this calibration equation, the limit of detection in concentration units was determined as

10 $$C_{LOD}=2.4\times 10^{-9}\;M$$

#### 3.2.2 Selectivity Tests

Figure 7a shows the sensor response (*T*_R_ %) and Figure 7b presents the selectivity results obtained on the LIG surface upon exposure to different analytes—ethanol, toluene, nitrotoluene, and DNT—at a concentration of 10^–3^ M. The resistance change for DNT was measured as 12.1%, while nitrotoluene, toluene, and ethanol induced changes of 7.25%, 4.51%, and 1.77%, respectively. Figure 7c shows the chemical structures of ethanol, toluene, nitrotoluene, and DNT. Ethanol is an alcohol consisting of a hydroxyl group (−OH) bonded to an ethyl group (C_2_H_5_). Toluene has a simple structure in which a methyl group (−CH_3_) is attached to a benzene ring. Nitrotoluene contains a nitro group (–NO_2_) attached to the benzene ring in addition to the methyl group (−CH_3_), whereas DNT bears two nitro groups at the 2- and 4-positions of the ring, along with the methyl group (−CH_3_). Each compound differs in its chemical structure and functional groups. In the selectivity tests, ethanol was used as a reference analyte to assess the sensor’s response to other analytes relative to the pure solvent.

**Figure 7:**
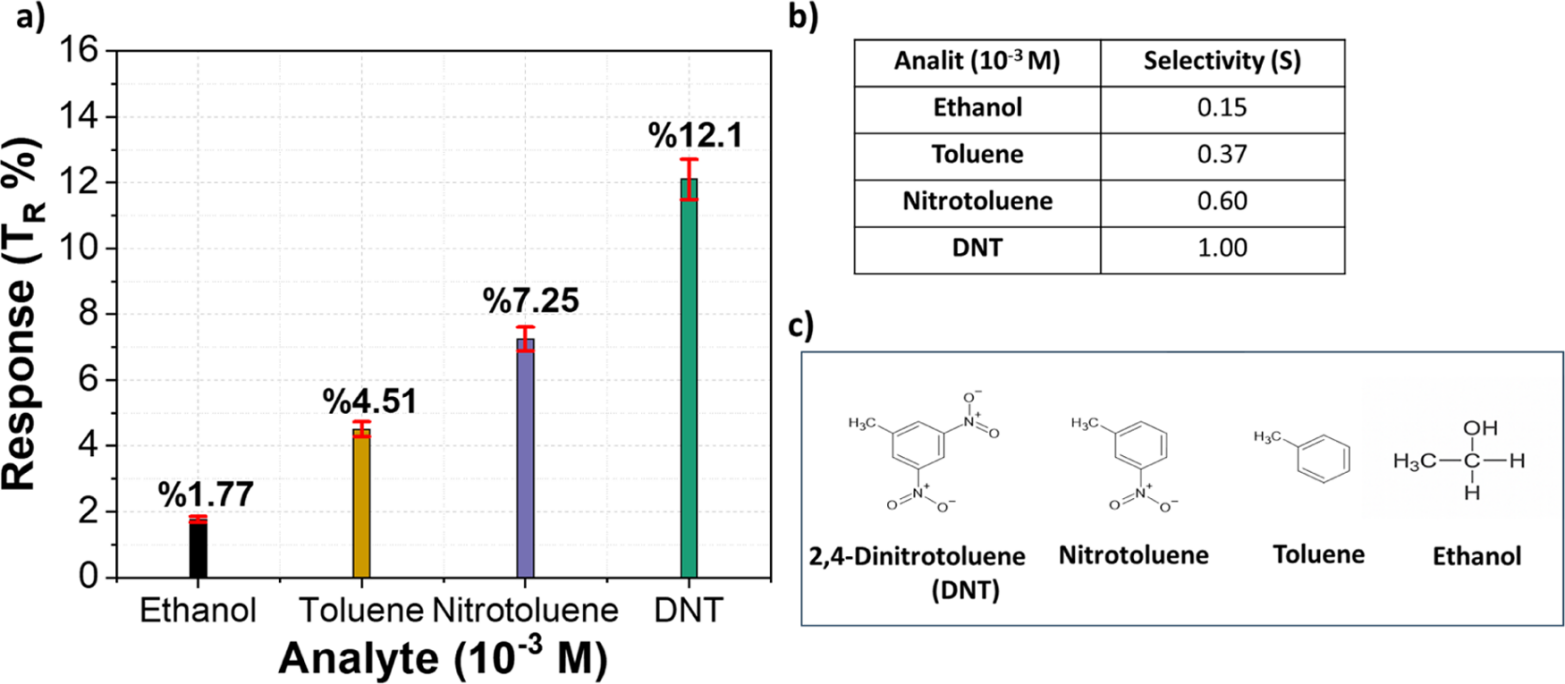
(a) Response of the LIG sensor toward DNT, (b) selectivity of the LIG sensor toward DNT compared with other analytes at 10^–3^ M, and (c) chemical structures of ethanol, toluene, nitrotoluene, and 2,4-dinitrotoluene (DNT).

The selectivity of the sensor, calculated using eq 6, revealed relative responses of 0.60, 0.37, and 0.15 for nitrotoluene, toluene, and ethanol with respect to DNT, respectively, confirming the strong specificity of the LIG-based sensor toward DNT. The noticeable response to nitrotoluene can be explained by its structural similarity to DNT, as both contain electron-withdrawing −NO_2_ groups that interact with the LIG surface through π–π stacking and charge-transfer processes. However, the stronger response of DNT is likely due to the presence of two nitro substituents, which enhance the electron-withdrawing effect and promote more efficient adsorption and charge transfer.

To ensure reproducibility, each data point in Figures 6 and 7 represents the average of at least three independent measurements, with error bars indicating the corresponding standard deviations. The low variability among replicates confirms the uniformity of the fabricated LIG films and highlights the robustness of the sensing platform.

## 4 Conclusion

In this study, a label-free chemiresistive sensor platform based on laser-induced graphene (LIG) was developed for the detection of 2,4-dinitrotoluene (DNT). The direct laser writing approach enabled the rapid and scalable fabrication of porous and conductive LIG films without the need for additional chemical modification or biorecognition elements. Electrical measurements confirmed the high conductivity of LIG, ensuring efficient electron transport, while XPS analysis verified DNT adsorption through the appearance of a nitro nitrogen (−NO_2_) peak as a distinct chemical signature.

The sensor’s analytical performance demonstrated a limit of detection (LOD) of 3.79% and a corresponding concentration limit of 2.4 × 10^–9^ M derived from the calibration curve. This result demonstrates the device’s high sensitivity in detecting DNT, even at low concentrations. Furthermore, selectivity tests revealed strong discrimination with superior response to DNT, consistent with the presence of two nitro groups that enhance adsorption and charge-transfer interactions.

Overall, this work demonstrates a simple, cost-effective, and scalable strategy for the direct detection of nitroaromatic explosives using unmodified LIG, highlighting the strong potential of LIG-based platforms for rapid, sensitive, and selective detection in security, defense, and environmental applications.
